# The cost of school holidays for children from low income families

**DOI:** 10.1177/0907568218779130

**Published:** 2018-05-31

**Authors:** Hilary Stewart, Nick Watson, Mhairi Campbell

**Affiliations:** University of Glasgow, UK

**Keywords:** Childcare, children, hunger, poverty, school holidays

## Abstract

School holidays can be stressful periods for children from low-income families. Poor provision of appropriate childcare, limited access to enrichment activities, and food insecurity mean that children’s health and well-being can suffer and their learning stagnate or decline. This article examines and documents the evidence that has emerged on this topic and aims to raise its profile and the impact on children’s lives. It makes the case for further academic scrutiny of this unexamined and neglected subject.

## Introduction

The summer holidays are often sentimentalized as a happy and carefree time for children, abound with new experiences and opportunities to play, relax, create memories, and develop essential social skills. While this is true for many children, for some, the school holidays are a stressful and impoverished period of isolation, boredom, and inactivity (Blazer, 2011). For low-income families, summer holidays often entail increased financial pressures, food insecurity, poor health, and exclusion from culturally enriching and healthful activities (Rai, 2015). Unequal access to and limited participation in such engaging activities means that many children miss out on interesting educational and developmental opportunities otherwise enjoyed by their more affluent peers (Blazer, 2011; Meyer et al., 2004; Summer Learning Association, 2009a). As a result, their learning stagnates and declines while their health and well-being also suffers (Graham et al., 2016; Slates et al., 2012). There is now an emerging body of evidence which suggests that the prolonged summer break has an accumulative effect on educational outcomes and may be one of the most fundamental, yet least acknowledged, contributors toward the attainment gap between richest and poorest children, accounting for almost two-thirds of the gap by the time children reach the age of 14 (Alexander et al., 2007, 2016; Slates et al., 2012; Von Drehle, 2010).

Despite ongoing commitment from successive UK governments to address child poverty and persistent attainment gaps (Carter-Wall and Whitfield, 2012), the impact of the school holidays on children’s educational development and well-being remains an under-explored topic, with ameliorative measures being provided by third-sector initiatives. Pressure groups such as Feeding America and End Summer Hunger in the United States and End Hunger UK in the United Kingdom have been established with the aim of both providing food over the summer but also to campaign and bring this issue to the forefront of public policy. There is, however, a relative dearth of academic research in this area and on holiday childcare more generally, despite its importance to families and the economy, and what little is available is largely gray literature (Cameron and Kiss, 2017; Graham et al., 2016). Presenting and engaging with the findings that have emerged from collaborative research into potential sustainable models of childcare, food, and learning opportunities for low-income families during the summer holidays, this article aims to raise the profile of these issues and their impact on children’s lives and make the case for further academic scrutiny of this topic. The effects of living in poverty on families and children are complex and multifactorial (Barnes et al., 2010). There are clearly broader structural inequalities at work in shaping educational outcomes and children’s well-being. However, the cost of summer holidays and its effect on attainment, both educationally and in terms of well-being, points to a policy blind spot with respect to providing equal opportunities for children.

This article examines the challenges families face during the school holidays and the effects that these cumulative disadvantages have on children’s educational attainment and well-being. This is the first time this research has been drawn together, looking across a range of issues including isolation, hunger, boredom, enrichment activities, childcare and their intersection with well-being and educational attainment. While focusing primarily on UK literature, it does draw on international evidence where appropriate. Electronic databases of peer-reviewed and gray literature, including Web of Science, Embase, Medline, ERIC, Open Grey, and Google Scholar, were searched using terms for school holiday (including “vacation,” “not in term time,” and “non term time”) and for childcare and nutrition (including “food poverty” and “food security”). The results, in combination with further iterative searches and articles from personal libraries, were used to develop this discussion. The article begins with a consideration of the effect of the summer holidays on children’s educational attainment and development by examining the phenomenon of *summer learning loss*, that is, the academic decline experienced by children as a result of the prolonged summer break and the disruption to learning that this entails (Alexander et al., 2007, 2016). We are not suggesting that this is the key reason for addresing this issue, rather it provides firm, empirical and objective evidence for the harm summer holidays can cause some children. The article then examines some of the problems that may be the cause of this learning loss, focusing in particular on the provision and cost of childcare over the holiday period before discussing the growing problem of food insecurity. The article concludes by arguing that there is a strong case for action in this area. These problems can be tackled, as evidence from the third sector demonstrates; what is required is the right investment and policy initiatives from government.

## Summer learning loss and the attainment gap

Research on *summer learning loss* shows that during the school holidays, children’s learning is not only at risk of stagnating but regressing, and that this decline may be more pronounced in children from low-income families, as well as for children with learning disabilities and children for whom English is a second language (Graham et al., 2012; Kerry and Davies, 1998). Some have argued that summer learning loss is the main factor behind the attainment gap between the richest and poorest students and that this is predominately driven by the inequalities experienced during the summer holidays (Alexander et al., 2016; Blazer, 2011).

Successive UK-based governments have made closing the attainment gap a key policy target, where across the country levels of inequality are greater than many other Organisation for Economic Co-operation and Development (OECD) countries (Andrews et al., 2017; Sosu and Ellis, 2014). The importance of this has been intensified by austerity where educational disadvantage has played a key role in shaping future outcomes (Carter-Wall and Whitfield, 2012; McCluskey, 2017). Recent governmental approaches in England have moved away from a more “broadly structural approach informed by social justice to a narrower focus on ‘troubled families’” (Ridge, 2013: 412). This approach relies heavily on the idea that educational outcomes can be improved through “raising aspirations” in low-income families (Carter-Wall and Whitfield, 2012). As a result, these approaches have emphasized individualized strategies of attitude and behavior modification, and focus on promoting resilience while neglecting more structural understandings of poverty and educational disadvantage (Carter-Wall and Whitfield, 2012). As such, recent poverty strategies have been criticized for treating children as prospective workers of the next generation who should be equipped with the tools to remediate their future disadvantage with little regard for childhood as an experience in and of itself (Ridge, 2013). Although this represents a valid concern regarding the nature of learning and ideological underpinning of children’s education, pragmatically speaking educational outcomes have real consequences for future life chances.

There has been little research into summer learning loss in the United Kingdom and therefore much of the literature in this area is North American in origin. However, in a recent survey of over 1000 primary and secondary schools in England, 77% of primary school leaders and 60% of secondary leaders voiced concern about summer learning loss among their students (Key Insights, 2014). Evidence for summer learning loss shows that during term-time, children across all income groups learn basic skills at similar rates; however, during the summer months, children from low-income families fall weeks or months behind their middle- and high-income peers (Alexander et al., 2007; Chaplin and Capizzano, 2006; Miller, 2007; Terzian et al., 2009; Von Drehle, 2010). This *backslide* in learning is usually measured using standardized tests administered before and after summer holidays (Cooper et al., 1996; Downey et al., 2004; Entwisle and Alexander, 1992; Heyns, 1978). Evidence submitted to the All-Party Parliamentary Group on Hunger (APPG) (Forsey, 2017) in the United Kingdom suggests that it takes up to 6 weeks for teachers to re-teach material forgotten over the summer period.

In their review of 39 studies on pre-secondary children’s learning during the summer period, Cooper et al. (1996) conducted a meta-analysis on the 13 highest quality studies and discovered that summer learning loss affects different subject areas to varying degrees. For example, learning loss was more substantial in mathematical computation and spelling skills than in reading. This is likely because these particular skills are based in factual and procedural knowledge and therefore more susceptible to forgetting if they are not reinforced through practice (Cooper et al., 1996). While all students regardless of income lost approximately equal amounts in maths skills (an average of 1.8 months of progress) and spelling skills (4 months of progress), low-income students are subject to the largest learning loss when it comes to reading recognition, losing approximately 1.5 months while higher-income children gained around 2.3 months (Cooper et al., 1996). The researchers found little evidence to suggest that students’ prior achievement levels, gender, or ethnicity had any consistent influence on summer learning loss; however, they did find that learning loss became more of an issue as children got older (Cooper et al., 1996).

Further evidence of summer learning loss comes from Alexander et al. (2007) who employed data from the Baltimore Beginning School study which followed a representative random sample of 790 school children from first grade until the age of 22. By analyzing data from reading comprehension tests given to the same students twice-yearly (autumn and spring), the researchers were able to isolate gains made during the school term from those made during the summer. When test scores reflected predominately term-time learning, low-income students kept pace with higher-income classmates (Alexander et al., 2007). During the summer however, higher-income students’ reading skills continued to improve while lower-income students lost ground. By the end of fifth grade (Primary 6 in Scotland/Year 5 in England), students from higher-income homes had gained approximately 47 points in their test scores thanks to their continued summer learning. For children from low-income homes, test scores decreased by 2 points over the same period. Alexander et al. (2007) concluded that by ninth grade (Secondary fourth year in Scotland/Year 10 in England), almost two-thirds of the achievement gap between higher- and lower-income children was explained by unequal access to summer learning opportunities during their early school years.

Downey et al. (2004) have reported similar findings after examining the learning rates of high- and low-income students over the school year and during the summer holidays. Using data from 20,000 children included in the Early Childhood Longitudinal Study, they found that the attainment gap was already present before school began and continued to widen after school started. However, the results indicated that once at school, the attainment gap between high- and low-income children grew much more rapidly over the summer months than it did during term-time (Downey et al., 2004). In the United States, this has prompted debate on how best to tackle summer learning loss, with reform of the school calendar implemented in some states to support Year Round Education (YRE) (Kerry and Davies, 1998). Others have argued that summer learning loss might be best addressed through the provision of high-quality summer camps and activities, a consideration which we will return to in our discussion of food insecurity (Graham, 2014).

What these studies highlight is that summer learning loss is cumulative in that low-income children fall further and further behind higher-income students year on year (Terzian et al., 2009). It is important to note that although we have focused on the issue of summer learning loss and the educational disadvantage this entails for poorer children, we would like to stress that lower-income families are not a homogeneous group and the relationship between poverty and educational attainment is inherently complex (Carter-Wall and Whitfield, 2012). While learning opportunities and enriching activities are clearly inhibited by cost, children’s learning and well-being is also mediated by many other activities.

That being said, there is strong evidence to suggest that children’s learning and well-being is put at risk without access to affordable, high-quality, and engaging childcare, the absence of which may also inhibit earning potential for parents (Diss and Jarvie, 2016). Children also require adequate food and other basic commodities during the holiday period. This article now turns to a discussion of some of the issues associated with holiday childcare for low-income families.

## Childcare

Accessing affordable and good-quality childcare during the summer months is essential for both the economy and families, enabling parents to continue with their ongoing commitments and responsibilities safe in the knowledge that their children are in a secure, fun, and engaging space (Cameron and Jarvie, 2016). Paid employment is politically endorsed as the route through which low-income families can remediate the poverty and inequality they experience, with childcare portrayed as the means of seizing these work opportunities (Petrie, 2015). However, holiday childcare costs are high and exceed many family budgets with 1 week of full-time care in the UK costing an average of £124.23 (Cameron and Kiss, 2017: 3). As Petrie (2015: 283) has recently noted, nursery places cost 77% more than they did in 2003 while earnings have remained largely unchanged. For many families, the high cost of childcare can mean that during the school holiday period, they are financially no better off in work, with some families seeing zero net income gain while other low-income earners can expect to take home as little as £1.96 per hour after having paid for childcare (Harding et al., 2017: 24; Petrie, 2015). A report by the Institute for Fiscal Studies showed that although the proportion of children with working parents rose between 2009-2010 and 2013-2014, a decrease in real earnings has meant reduced incomes for families and therefore the proportion of children in poverty from working families increased from 54% to 63% over the same period (Belfield et al., 2015). More recently, Child Poverty Action Group (CPAG) (2017a) reported that the number of children living in poverty is now 4 million and that in-work poverty is the most prevalent form of child poverty with 67% of poor children living in low-income households.

A Joseph Rowntree report into the potential of parental employment to reduce child poverty found increased exits from work from July to September which the researchers attributed to parents’ inability to source and afford childcare during the summer months (Simmonds and Bivand, 2008). The report also showed increases in lone parent benefit claims during the summer holidays, indicating that this period was particularly difficult for lone parents to sustain work and confined many parents with caring responsibilities to a “no-pay, low-pay cycle” (Rabindrakumar, 2014, 2015; Simmonds and Bivand, 2008: 16). As CPAG (2017a) has noted, 47% of children from lone parent families live in poverty. These findings have been further supported by research from Graham and McQuaid (2014) who reported that the lack of suitable and affordable childcare acts as a significant barrier to employment for lone parents. Of particular concern here is the risk of benefit sanctioning as lone parents are increasingly obliged to fulfill work-related requirements and actively seek work which may be especially difficult to accommodate during the summer without appropriate childcare (Graham and McQuaid, 2014; Petrie, 2015). Moreover, without access to suitable childcare and when faced with these sanctions or disciplinary procedures at work, some parents are forced to leave their children in risky situations with minimal supervision or indeed home alone (Petrie, 2015).

Literature exploring the lack of childcare during the summer holidays is limited, even though this is regularly raised whenever childcare problems are discussed. Under the Childcare Act 2006, local authorities in England and Wales are legally obliged to provide adequate childcare to parents who are working or undertaking training for work and to audit parental demand (Cameron and Jarvie, 2016; Rutter, 2015). Since September 2016, parents and childcare providers in England have had the “right to request” that local schools be used to provide wraparound and holiday childcare (Cameron and Jarvie, 2016: 20). This policy was implemented to encourage schools to make better use of existing facilities and has the potential to fill holiday childcare gaps through offering families accessible and affordable childcare in familiar settings. This seems to represent a renewed interest in the potential of *Extended Schools*, a scheme originally introduced by New Labour to provide a range of services in local schools such as childcare, recreational activities, and parental support services but which quickly dissipated following austerity cuts and the end of ring-fenced funding (Cameron and Jarvie, 2016). However, unless parents are openly informed of their rights to request and the process involved and until this right is statutorily enforced, this scheme can act only as a means of encouraging schools to consider such provision (Cameron and Jarvie, 2016). In England, some local authorities have reported that the policy has been difficult to realize as a result of limited resources and the pressures and demands surrounding the implementation of the new entitlement to a minimum of 30 hours childcare (Cameron and Kiss, 2017). As of July 2017, the *right to request* policy has had little impact and would benefit from local communication drives which clarified the process as well as being statutorily implemented to ensure that these requests are properly investigated (Cameron and Kiss, 2017).

In contrast to England and Wales, although Scottish local authorities must take a strategic approach toward providing accessible childcare, there is no legal obligation for them to do so and therefore no statutory guidance or legal definitions regarding “sufficiency” of provision (Cameron and Jarvie, 2016). Recent research on the cost of school holidays and childcare in Glasgow found that 86% of parents agreed that childcare is too expensive and a further 57% reported that childcare did not suit their working schedule (CPAG, 2015: 42). According to the Holiday Childcare Survey for 2015, only three local authorities in Scotland had enough holiday childcare for working parents (Rutter, 2015).

Shortages in provision have worsened across the United Kingdom in recent years with more than 5 million children living in areas where there is inadequate childcare (Cameron and Jarvie, 2016). This is a particular problem for families living in deprived areas where there is less provision generally and has been exacerbated by the closure of over 100 *Sure Start* centers which are disproportionately located in poorer areas (Rutter and Stocker, 2014). Holiday support for children with Additional Support Needs and disabilities is particularly poor, with local authorities reporting little to no provision for such families (Cameron and Jarvie, 2016). Many parents feel that this lack of suitable care disrupts routine and that limited access to structured activities during the holiday period negatively impacts their children’s well-being (CPAG, 2015). Provision for older children, especially children aged between 11 and 14 is also sparse and although parents often felt that children this age were still too young to be left unattended for too long, many childcare providers do not cater to children aged 11 and over (Rabindrakumar, 2014).

For some parents, the difficulties in managing childcare costs and scheduling have resulted in them taking unpaid leave, choosing zero-hour contracts or self-employment and to shoulder the risks and low pay associated with these instead of taking on more permanent, better rewarded, and secure employment (Hawkins, 2014; Hoggart and Vegeris, 2008; Rabindrakumar, 2014). Following a survey of 614 lone parents on the difficulties of accessing suitable childcare during the holidays, Hawkins (2014) found that 29% had been forced to reduce their working hours, and 16% had stopped looking for work altogether or turned down employment in order to care for their children. The Holiday Childcare Survey for 2014 found that some parents reported their employers were often unsympathetic and inflexible when it came to scheduling work around childcare needs, leading 17% of parents to admit to taking sick days in order to look after their children (Rutter and Lugton, 2014: 3). If this pattern of absence is applied more generally to all UK parents, “one day missed to cover childcare every year represents over 900,000 lost working days and costs the UK economy nearly £100 million every year” (Rutter and Lugton, 2014: 3).

The current UK Conservative government has promised to double the provision of free childcare from 15 to 30 hours for children aged 3 and 4 from September 2017; however, this will only be available to working parents in England (with similar policies planned in future for Scotland and Wales) who earn the equivalent of 16 hours per week at national living wage (Johnes and Hutchinson, 2016). This restricted eligibility means that children coming from families on the lowest incomes or from non-working households miss out (especially where parents may be on zero-hour contracts or in insecure employment) and is particularly troubling given that it is this group of children who stand to gain the most from good-quality, early years education (Johnes and Hutchinson, 2016; Preston, 2008). There are also concerns that given the current shortage and patchy provision of nursery places, the government budget to implement this initiative is not sufficient and that the extra costs needed to subsidize this funding shortfall will be passed onto parents (Johnes and Hutchinson, 2016). What is more, Johnes and Hutchinson (2016) have warned that the increased competition for limited nursery places means there is a danger that childcare providers will prioritize children who qualify for the full 30 hours of care at the expense of those who only qualify for the universal 15 hours. This policy has therefore been criticized for prioritizing parental employment over children’s development and overlooks the opportunity to implement high-quality early years education and address attainment gaps (Johnes and Hutchinson, 2016). Since the free childcare is only available during school term-time, the problem of finding care for children outside term-time remains, even for parents able to access the free childcare.

What our review of childcare demonstrates is that provision of holiday care is not only expensive but unsuitable and insufficient to meet the needs of low-income families. While childcare is portrayed as a service which facilitates parental employment and by extension the amelioration of poverty, the issue of childcare as a positive experience which supports children’s developmental needs and well-being is overlooked (Petrie, 2015). We now turn to a discussion of the growing problem of food insecurity during the summer holidays and the impact this has on children’s well-being and education.

## Food insecurity

Sufficient access to nutritiously adequate food is recognized as a basic human right yet there are many low-income families in the United Kingdom who struggle to get enough food and are classified as *food-insecure* (Defeyter et al., 2015; Graham et al., 2016). Food insecurity is typically defined as insufficient and insecure access to nutritionally adequate food due to resource constraints. It has become a topic of growing concern amid rising demands for food aid and the increased usage of food banks across the United Kingdom (Graham et al., 2016; Taylor and Loopstra, 2016). According to data on food insecurity in the United Kingdom gathered by United Nations, in 2014, an estimated 8.4 million people lived in households with insufficient food security (Taylor and Loopstra, 2016). However, without any national monitoring of food insecurity, Taylor and Loopstra (2016) warn that the real magnitude of this problem remains largely obscured and allows the government to remain inactive on the issue (Rai, 2015).

Recent research suggests that low-income families find the long summer holidays particularly difficult as children lose access to free school meals and experience food insecurity or what has been referred to as “holiday hunger” (Graham et al., 2016: 2; Gill and Sharma, 2004). A recent report by the APPG in the United Kingdom (Forsey, 2017: 4) estimates that up to 3 million children are at risk of going hungry during the school holidays. Although children who qualify for free school meals through low income are identified as those at greatest risk from holiday hunger, they account for only a third of this figure, highlighting the food insecurity faced by families experiencing in-work poverty (Forsey, 2017: 11).

Oral evidence submitted by researchers and food bank staff to the APPG described how food bank use accelerates significantly among families during the long summer holidays as they struggle to feed their children every day (Forsey, 2017: 9). Last year, Britain’s largest food bank network, *The Trussell Trust*, found that the number of families seeking food aid because they could not afford to feed their children over the school holidays had doubled since 2015 with 5000 extra food parcels going to children in July and August than in the preceding few months (Forsey, 2017: 13). In addition to relying on food banks, several pieces of research have reported that many parents have resorted to skipping meals as a “coping strategy” in a bid to feed their children over the summer holidays (CPAG, 2017b, 2015; Forsey, 2017: 16; Graham et al., 2016).

The malnourishment that comes from food insecurity can manifest in children’s bodies in a number of ways. Upon return to school, teachers have observed the obvious physical consequences in children who are “noticeably thinner” and sluggish as well as the negative effects it exacts on their mental well-being and ability to learn (Forsey, 2017; Rai, 2015: 3). Malnutrition renders children unalert and unable to concentrate, demonstrating how holiday hunger can also exacerbate summer learning loss with children who do not get enough to eat over the summer period returning to school “weeks, if not months, intellectually behind their more fortunate peers” (Forsey, 2017: 23). A recent report commissioned by CPAG (2017b: 06) in collaboration with the Royal College of Paediatrics and Child Health (RCPCH) found that of the 266 pediatricians surveyed, 253 believed that food insecurity contributed to the ill-health of the children they treated. The inability to provide nutritionally adequate food was associated with both poor growth and rising obesity in deprived children and that some parents struggled to afford essentials such as clothes, toothpaste, and toothbrushes (CPAG, 2017b: 06). Of the 266 pediatricians surveyed by CPAG (2017b: 11), 251 believed that the worry and stress associated with poverty negatively harmed the psychological well-being of children, revealing the less obvious manifestation of material disadvantage in feelings of shame, stigma, and anxiety (Ridge, 2013). These combine with and reinforce the harm caused by malnutrition and Hirsch (2013; CPAG, 2017b) has estimated that child poverty costs the country £1.5 billion a year as a result of the increased need for acute healthcare.

That children are being left to go hungry at all is abhorrent but is all the more worrying given the effect that this malnutrition has on children’s learning and attainment as “Children who are hungry more frequently fall behind academically” (Forsey, 2017: 25). While all children experience a gap in formal educational instruction during the holidays, it is children with impoverished diets and with limited access to enriching activities who are most prone to learning loss. It has been estimated that it takes up to 6 weeks for such children to physically and mentally readjust and be ready to reengage with the school curriculum (Forsey, 2017).

Although hunger represents a significant barrier to children’s education and well-being, the cumulative struggles facing low-income families during the holidays such as the limiting costs of activities and suitable childcare demonstrate the multiple disadvantages that children from food-insecure homes experience (CPAG, 2015; Forsey, 2017). The APPG report identified that among the many factors associated with increased risk of food insecurity (such as high living costs, low incomes, insecure employment, and delayed benefit payments and sanctions) were three unique contributors: the increased overall cost of looking after children during the holidays, the lack of affordable childcare, and the loss of free school meals (Forsey, 2017). Addressing holiday hunger is therefore about much more than the provision of affordable and nutritious food and forms part of a much bigger picture in terms of children’s well-being, development, and life chances (CPAG, 2015; Forsey, 2017). As we have already highlighted, children from lower-income families are less likely to take part in enrichment activities during the summer holidays (Cooper et al., 1996). As such, initiatives which center around the provision of fun, enriching activities, as well as nutritious food, have been suggested as a means of ameliorating the multiple challenges facing low-income families in a way that regards the interests of children and avoids the stigma of “feeding stations for the poor” (CPAG in Forsey, 2017: 51; Graham, 2014; Ridge, 2013).

This is supported by research from Graham (2014) who argues that inequality gaps experienced by low-income families may be addressed through summer activity programs which maintain academic skills through a blend of learning, sport, and enrichment activities coupled with with community food provision. According to Cooper et al. (1996), these sorts of programs would not only address educational attainment but also promote interpersonal skills and promote wellbeing. Feedback from third-sector food and fun programs report positive impacts upon children’s diet, social inclusion, physical activity, and learning opportunities as well as increased levels of concentration, confidence, and skills (Defeyter et al., 2015; Forsey, 2017). Moreover, the various opportunities for parents to participate, volunteer, and access nutritional food and activities bolstered parental confidence, improved healthy food awareness, and meal preparation skills as well as offering informal social support though community networks (Defeyter et al., 2015; Forsey, 2017; Graham et al., 2016). These evaluations of summer programs provide valuable insights into how projects can make best use of existing facilities and local resources and suggest that a comprehensive system of protection against the challenges of poverty during the summer months could be implemented with the right funding and government support (Forsey, 2017).

The APPG hunger report has urged the UK government to eradicate holiday hunger by enacting a statutory requirement of local authorities to facilitate and coordinate summer food and fun programs (Forsey, 2017). Recommendations presented in the report draw upon examples of good practice found in many summer food and fun projects which have so far been successfully provided by third-sector initiatives (Forsey, 2017). Despite growing evidence on the ills of food insecurity, there has been no strategic commitment from the UK government or legal requirements for local authorities to implement summer programs to tackle holiday hunger (Forsey, 2017; Gill and Sharma, 2004). This is not an issue which can be left to the benevolence of volunteers and third-sector provision and must become a policy priority of the government.

## Conclusion

A clear conclusion that can be drawn from the literature is that for many low-income families, the summer holidays are an exceptionally difficult period. Not only are children from poorer families prevented from participating in enriching activities that many others take for granted, but their health is also put at risk through malnourishment, isolation, and extended periods of inactivity. These disadvantages emerge from a range of overlapping issues, including low income; welfare cuts and unsuitable, inaccessible, and insufficient childcare; the absence of free school meals; and the lack of suitable childcare during the summer. Parents’ opportunities to pursue working commitments are made increasingly difficult and this may further hinder attempts to move out of poverty. The disadvantages that low-income families face meld together and reinforce each other in ways which deeply permeate children’s lives (Petrie, 2015; Ridge, 2013). As we have shown, there is evidence to suggest that the combination of these factors exacts a toll upon children’s learning through the phenomenon of summer learning loss which in turn may contribute to the widening of attainment gaps between economically advantaged and disadvantaged students (Alexander et al., 2007; 2016).

Although presently there is no definitive solution to the problems faced by low-income families, evidence suggests that if we are to tackle learning inequalities and support children’s education, health, and well-being, a system of social protection is required to negate the impact of poverty during the summer holidays. While ideally these problems would be resolved through a more up-stream, structural approach tackling what Marmot (2005) calls the “cause of the causes,” such an approach is likely to take time. These children need help immediately. The literature indicates that there are a number of key areas which must be addressed. First and foremost, steps must be taken to address the national problem of food insecurity to ensure that children do not go hungry or become malnourished during the school holidays. Not only does holiday hunger jeopardize children’s physical and mental well-being, it also impedes their capacity to participate in activities and learn (Forsey, 2017). Second, providing accessible, good-quality childcare which meets the diverse needs of families is vital if children’s learning and well-being is to be supported while enabling parents to pursue better paid and more secure employment. Third, although there is a substantial amount of evidence to support the claim that summer learning loss is a problem and a particular concern for low-income families, there is a lack of research within the UK context and on the long-term impact upon attainment and life outcomes, a gap which must be addressed through rigorous academic scrutiny.

However, given the already extensive and overwhelming evidence on the harm that surrounds the summer holiday experiences of children from low-income families, what is needed now is not just more research on the extent or nature of these inequities; rather there needs to be an emphasis on research on interventions that seek to tackle these inequalities. We need to know what works and what does not in helping to improve outcomes for children from low-income families over the summer and how best can we reform and change the lives of these children. Unless we take steps to tackle this problem, the evidence would suggest that attempts to rectify the attainment gaps in education, health, and well-being that exist between the wealthiest and poorest school children will be unlikely to succeed.
